# Age-Stratified QTL Genome Scan Analyses for Anthropometric Measures

**DOI:** 10.1186/1471-2156-4-S1-S31

**Published:** 2003-12-31

**Authors:** Stephanie R Beck, W Mark Brown, Adrienne H Williams, June Pierce, Stephen S Rich, Carl D Langefeld

**Affiliations:** 1 Department of Public Health Sciences, Wake Forest University School of Medicine, Winston-Salem, North Carolina, USA

## Abstract

With the availability of longitudinal data, age-specific (stratified) or age-adjusted genetic analyses have the potential to localize different putative trait influencing loci. If age does not influence the locus-specific penetrance function within the range examined, age-stratified analyses will tend to yield comparable results for an individual trait. However, age-stratified results should vary across age strata when the locus-specific penetrance function is age dependent. In this paper, age-stratified and age-adjusted quantitative trait loci (QTL) linkage analyses were contrasted for height, weight, body mass index (BMI), and systolic blood pressure on a subset of the Framingham Heart Study. The strata comprised individuals with data present in each of three age groups: 31–49, 50–60, 61–79. Genome-wide QTL analyses were performed using SOLAR. Over all ages, a linkage signal for height was detected on chromosome 14q11.2 near marker GATA74E02A (LOD for ages 31–49 = 2.38, LOD for ages 50–60 = 1.84, LOD for ages 61–79 = 2.45). Evidence of linkage to BMI in the 31–49 age group was found on chromosome 3q22 (GATA3C02, LOD = 2.89, *p *= 0.0003) at the same location as the signal for weight (LOD = 3.10, *p *= 0.0002). Linkage was also supported on chromosome 1p22.1 for BMI (LOD = 2.21, *p *= 0.0014) and weight (LOD = 2.47, *p *= 0.0007) in the 31–49 age group. Our age-stratified results suggest that QTL that are expressed over long periods of time and affecting multiple, correlated traits may be identified using genome scan and variance-component methodology to help detect early and/or late gene expression.

## Background

While it has long been known that age is a major risk factor for cardiovascular disease (CVD), it is unclear how to best capitalize upon this source of variation in the search for CVD-predisposing genes. In particular, identifying those strategies that might take advantage of a penetrance function (probability of affection given a genotype) for a disease that varies with age. Although parametric linkage analysis models have incorporated age-specific penetrance in the likelihood calculations, nonparametric linkage analysis has not. Similarly, methods of analysis for quantitative traits (e.g., variance-component methods) typically incorporate age as a covariate in analytic models. Franklin et al. [1] report a shift in favor of pulse pressure (PP) and systolic blood pressure (SBP) as significant predictors of coronary heart disease (CHD) risk at age 60 and above, while diastolic blood pressure is a stronger predictor of CHD while under 50. Between the ages 50 and 60, all are similarly predictive. This suggests that within individuals the expressivity of these moderately heritable traits may be dependent upon age. However, not all traits are influenced by measured covariates. For example, height is likely not age-dependent between ages 20 and 50. More importantly, the genes which influence a phenotype late in life may not have an influence early in life and vice versa.

In this report, the Framingham Heart Study (FHS) longitudinal data collection scheme and genome scan data are used to explore linkage signals for selected quantitative trait loci (QTL) over time. The traits of interest (height, weight, body mass index (BMI) and SBP) are used within specific time windows to develop a better understanding of the penetrance of a disease over time. Since height varies modestly between the ages of 30 and 80, it is used as a contrast to traits that likely exhibit age-dependent environmental and genetic influences. In addition, these analyses may help identify regions of the genome that may harbor QTL.

## Methods

The FHS recruited a total of 10,333 individuals from two cohorts to participate in research whose aim was to monitor and identify common factors related to CVD. The original cohort (Cohort) led to recruitment of a second cohort (Offspring) in which spouses and children of the Cohort were ascertained. Over the years the study enrolled 1644 spouse pairs, 2616 offspring, and 34 stepchildren. The most informative 330 pedigrees were submitted for genome scan to the NHLBI-supported Mammalian Genotyping Service (MGS), which typed 428 markers with an average distance between markers of approximately 8.4 cM. The pedigrees contained 4692 individuals, of which 2885 had DNA for genetic studies. It is from this sample that our sample was constructed. Our sample required presence of both genotypic and phenotypic data from three age categories centered at 40, 55, and 70 years for each outcome and relevant covariates. These age categories included the nearest visit ± 9 years for ages 40 and 70 years; for age 55, an interval of ± 5 years was chosen. Uneven age categories were required to maximize the number of individuals that have data in all three intervals. In addition, it is important that the different intervals span meaningfully different periods of life. As a result of our sampling strategy, the individuals used in the analyses may vary between traits but not within a trait. Specifically, individuals in the BMI analysis may not have been included in the SBP analyses, but the same individuals are used for each of the BMI analyses. For those individuals in the age-stratified analysis, data from visit 7 and 3 from the Cohort and Offspring, respectively, were used in the age-adjusted analysis. An additional sample was constructed by including data for a subject from a family in the sample who was excluded from the original analyses due to death before age 61. QTL analyses for each trait were performed on the age group 40 data with the selected covariates for each trait as well as adjusting for early death of an individual and their age of death.

QTL multipoint variance-component analyses were then performed using Sequential Oligogenic Linkage Analysis Routines (SOLAR) [2] at each age category with marker allele frequencies being determined based upon the FHS sample data. The maximum likelihood method for hypothesis testing was performed using the relationship of identity-by-descent (IBD) statistics between family members and genetic covariance. The full model (QTL effect accounting for a portion of the genetic variance) with the null model (no QTL effect). In our analyses, only *a priori *covariates were considered and they were directly incorporated into the variance-component models with SOLAR [3].

Traits for analysis included SBP, BMI, weight, and height. In the Cohort, per FHS protocol, height was collected during selected visits only. Height was inferred for each participant using the previous height for visits in which a participant attended, but this data was not collected (for missing at the initial visit, height was inferred for each time point using the first recorded measurement). Where appropriate, traits were transformed to better approximate the distributional assumptions of conditional normality and homogeneity of variance. Collinearity and influence were examined using standard linear regression diagnostics [4]. These analyses suggested that height did not require any transformation but SBP, weight, and BMI were log-transformed. Weight was analyzed after adjusting for gender and height. SBP was analyzed after adjusting for gender and BMI. BMI and height were analyzed after adjusting for gender. For those individuals in the age-stratified analyses, data from FHS visit 7 and 3 from the Cohort and Offspring, respectively, were used in the age-adjusted analysis. An additional sample was constructed by including data for a subject from a family in the sample who was excluded from the original analyses due to death before age 61. Similar QTL analyses for each trait were performed on the age group 40 data with the selected covariates for each trait as well as adjusting for early death of an individual and their age of death.

## Results

The basic characteristics on the collective sample of study subjects, for both the age-stratified and age-adjusted analyses, are summarized in Table 1. For the traits of interest, participants, as they aged, were of shorter stature (height: 65.2 in, 65.0 in, 64.3 in) and more obese (BMI: 25.6 kg/m^2^, 26.4 kg/m^2^, 27.1 kg/m^2^). Blood pressure increased over time (SBP: 125.8 mm Hg, 131.7 mm Hg, 139.9 mm Hg). The mean age at the last exam attended before death was 71.8 (SD = 6.3, *N *= 229).

Using the genome scan data, the results for each age group are presented for the selected phenotypes. The regions presented in the tables reflect regions that provide some support in at least one of the three age categories or the age-adjusted analysis with multipoint LOD > 1.0. The results for height adjusted by gender appear in Table 2. The strongest evidence of linkage to height was at 14q.

Linkage to height was consistent for GATA74E02A (D14S742) on the distal part of 14p (LOD = 2.38 in the 31–49 age group, LOD = 1.84 in the 50–60 age group, and LOD = 2.45 in the 61–70 age group). The weakest evidence for linkage at this marker is in the age-adjusted analysis (LOD = 1.4508). No other marker had consistently strong evidence of linkage for height (LOD > 2) in any category. It is interesting to note that the modest evidence for linkage at 12q (LOD = 1.03) coincides with the region reported by Xu et al. [5].

The results for weight adjusted for height and gender are shown in Table 3. The strongest evidence for linkage occurs in the youngest age group (31–49), with LOD = 3.10 for GATA3C02 (D3S1744) and LOD = 2.47 for ATA2E04 (D1S1588). Evidence for linkage at these markers significantly decreases with age, as no other marker provides strong (LOD > 2) evidence in the older age groups. The results for BMI (adjusted for gender) are shown in Table 4. Similar to the results for weight, the strongest evidence for linkage occurs in the youngest group (31–45) with the same markers (GATA3C02, LOD = 2.89, D3S1744; ATA2E04, LOD = 2.21, D1S1588). Also, no other marker provided strong evidence for linkage (LOD > 2). These results suggest a hypothesis that these regions may harbor loci that influence obesity and that they may more easily be detected in relatively younger samples.

The strongest evidence for linkage in the age-stratified analyses of SBP occurs near ATA82B02 in age group 61–79 (LOD = 1.10) and GATA134B03 (D5S2845) in age group 50–60 (LOD = 1.01). It is interesting to note that these regions of interest showed virtually no evidence for linkage in other age groups. In the age-adjusted analysis, marker GATA188F04 (D21S2055) shows evidence for linkage on 21q (LOD = 1.21). However, there was no evidence for linkage in this region in the age-stratified analysis.

## Discussion

The age-stratified analyses explored in this study are restricted to a few cardiovascular- and anthropometric-related traits, which may exhibit age-dependent (e.g., BMI) and age-independent (e.g., height) penetrances. These analyses provide an interesting contrast in study design over that of an age-adjusted analysis from a specific clinic visit. The results from the age-stratified analyses for one potentially age-dependent trait, BMI, yielded larger maximum LOD scores in the younger stratum than the older stratum or age-adjusted analysis. The reduced evidence for linkage in the latter analyses may reflect the increasing influences of environmental factors or different genes working during different stages of life. The age-stratified and age-adjusted analyses tended to yield more similar maximum LOD scores for the likely age-independent trait, height.

Given the sample selection criteria, subjects were excluded from our original analyses who may have exhibited greater severity of BMI, weight, and SBP. In looking at causes of morbidity and mortality, Kim et al. [6] showed a positive linear relationship between CHD and BMI. Censoring of data due to morbidity and mortality from changing risk factor distribution may have occurred, affecting the contribution of those at the extremes of the phenotypic distribution. The impacts of these fatalities were examined, but neither height, weight, BMI, nor SBP exhibited any significant evidence for linkage.

Garrison et al. [7] showed smoking to be a potential confounder of the relationship between obesity and mortality. Additional analyses were computed adjusting SBP for smoking status (i.e., yes, no), since smoking status is positively correlated with SBP. However, no substantial differences in the linkage results were observed.

While this examination of the data yielded no significant results on chromosome 6 for BMI, which has been previously reported by Atwood et al. [8]. At least three reasons may contribute to this result. First, our sampling scheme was more restrictive. Second, in the sample employed in our analyses, BMI was log-transformed to better approximate distributional assumptions. Third, the age-stratified analysis presented here does not provide for a cohort adjustment. Such adjustment may minimize the generational differences in exercise, eating habits, medical care, etc., that has varied over the decades.

## Conclusions

**Figure 1:**
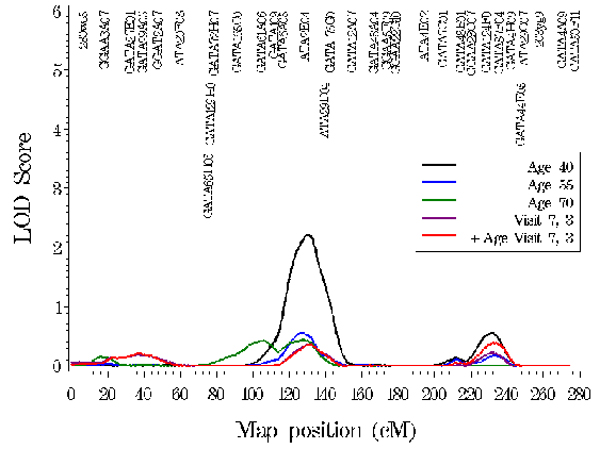
Chromosome 1 genome scan for BMI adjusted by gender.

**Figure 2:**
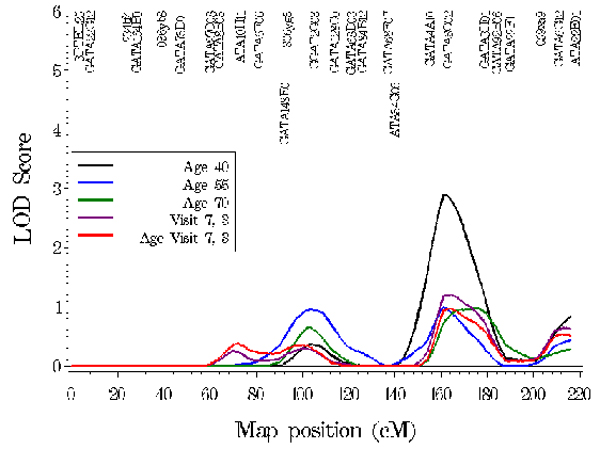
Chromosome 3 genome scan for BMI adjusted by gender.

When taking into account the many changes that occur within the human body throughout life, it seems highly plausible that variation in a phenotype with some genetic component may be affected by different genetic loci at different times. Given this scenario, an age-adjusted analysis would not necessarily optimally define these regions. While the correlation in the different age-stratified genome scans can be observed in Figures 1 and 2, there are also differences in the magnitude of the LOD scores at 1p22.1 and 3q22 for weight and BMI. The change in BMI from age group 40 to 55 and 70 is the only source of variation between the scans of these age groups. Age-stratified analyses may help to better identify regions which play a role in determining early and/or late gene expression or those which are constantly expressed. Thus, age-adjusted and age-stratified analysis plans provide complementary tools in the search for genes which influence traits.

## Figures and Tables

**Table 1:** Demographic distribution of traits with selected covariates^A^

	Ages 31–49	Ages 50–60	Ages 61–70	Age-Adjusted Cohort Visit 7 Offspring Visit 3
Age	(3.3, 795)	(1.0, 795)	(3.2, 795)	(5.7, 781)
Female	(442)	(442)	(442)	(442)
Height (in)	(3.8, 792)	(3.7, 792)	(3.7, 792)	(3.7, 778)
Weight (lbs)	(29.4, 795)	(25.9, 795)	(31.3, 795)	(30.5, 765)
BMI (kg/m^2^)	(3.7, 792)	(3.9, 792)	(4.4, 792)	(4.0, 762)
SBP (mm Hg)	(16.2, 795)	(18.5, 795)	(20.6, 795)	(18.2, 768)
Smoke	(777)	(795)	(793)	(767)

^A^Data are listed in the format mean (SD) for continuous traits or % (*N*) for dichotomous traits.

**Table 2:** Genome-wide scan results for height adjusted by gender.

		AGE 40		AGE 55		AGE 70		Age-Adjusted Cohort Visit 7 Offspring Visit 3	
		
Location	Nearest Marker	Highest LOD	LOD-1 Interval (cM)	Highest LOD	LOD-1 Interval	Highest LOD	LOD-1 Interval (cM)	Highest LOD	LOD-1 Interval (cM)
2p25.3	GATA165C07	1.52	0–13	1.14	0–16	0.88	0–20	0.94	0–22
12q13.13	GGAT2G06	0.70	36–96	1.03	0–104	0.62	36–102	1.05	36–114
14q11.2	GATA74E02A	2.38	0–22	1.84	0–24	2.45	0–27	1.45	0–24
19p13.3	GATA21G05	0.48	0–56	1.03	0–101	1.48	0–56	0.48	0–44

**Table 3:** Genome-wide scan results for weight adjusted by gender and height.

		AGE 40		AGE 55		AGE 70		Age-Adjusted Cohort Visit 7 Offspring Visit 3	
		
Location	Nearest Marker	Highest LOD	LOD-1 Interval (cM)	Highest LOD	LOD-1 Interval (cM)	Highest LOD	LOD-1 Interval (cM)	Highest LOD	LOD-1 Interval (cM)
1p22.1	ATA2E04	2.47	119–140	0.85	98–170	0.54	114–148	0.58	106–150
3q22	GATA3C02	3.10	155–171	1.17	146–180	0.99	148–200	1.03	152–188
6p21.2	GTAT1H06	1.12	18–90	0.94	20–98	0.34	26–64	1.65	29–61
8p21.3	COS140D4	1.38	24–64	1.49	24–63	0.90	18–80	1.08	12–64
12p13.32	GATA4H03	0.37	0–30	1.77	0–15	0.88	0–32	1.02	0–70
17q25.3	AFM044xg3	1.68	101–126	0.91	82–126	0.71	74–116	0.35	88–126
18p11.31	GATA88A12	1.70	0–44	1.36	0–46	0.40	0–34	1.08	0–42

**Table 4:** Genome-wide scan results for BMI adjusted by gender.

		AGE 40		AGE 55		AGE 70		Age-Adjusted Cohort Visit 7 Offspring Visit 3	
		
Location	Nearest Marker	Highest LOD	LOD-1 Interval (cM)	Highest LOD	LOD-1 Interval (cM)	Highest LOD	LOD-1 Interval (cM)	Highest LOD	LOD-1 Interval (cM)
1p22.1	ATA2E04	2.21	119–41	0.56	96–152	0.44	68–146	0.37	108–150
3q22	GATA3C02	2.89	156–175	0.95	70–138	0.97	150–200	0.96	150–190
8p21.3	COS140D4	1.45	42–63	1.51	24–62	0.87	18–78	1.02	10–64
12p13.32	GATA4H03	0.60	0–30	1.85	0–14	0.85	0–30	1.07	0–52
17q25.3	AFM044xg3	1.58	100–126	0.90	80–126	0.75	72–116	0.38	86–126
18p11.31	GATA88A12	1.74	0–43	1.34	0–46	0.49	0–34	1.14	0–38

**Table 5:** Genome-wide scan results for SBP adjusted by gender and BMI.

		AGE 40		AGE 55		AGE 70		Age-Adjusted Cohort Visit 7 Offspring Visit 3	
		
Location	Nearest Marker	Highest LOD	LOD-1 Interval (cM)	Highest LOD	LOD-1 Interval (cM)	Highest LOD	LOD-1 Interval (cM)	Highest LOD	LOD-1 Interval (cM)
5p14.3	GATA134B03	0.02	32–42	1.01	0–44	0.27	14–54	0.53	0–70
18q22.3	ATA82B02	0.00	-^A^	0.00	106–108	1.10	74–116	0.0000	0–102
21q22.2	GATA188F04	0.00	22–26	0.66	-	0.04	16–28	1.21	22–58

^A^-, denotes the entire length of the genotyped chromosome.
